# Improving the sensitivity of sample clustering by leveraging gene co-expression networks in variable selection

**DOI:** 10.1186/1471-2105-15-153

**Published:** 2014-05-20

**Authors:** Zixing Wang, F Anthony San Lucas, Peng Qiu, Yin Liu

**Affiliations:** 1 Department of Neurobiology and Anatomy, University of Texas Health Science Center at Houston, Houston, Texas, USA; 2 Department of Epidemiology, University of Texas MD Anderson Cancer Center, Houston, Texas, USA; 3 Department of Biomedical Engineering, Georgia Institute of Technology and Emory University, Atlanta, Georgia, USA; 4 University of Texas Graduate School of Biomedical Sciences, Houston, Texas, USA

**Keywords:** Variable selection, Gene co-expression network, Sample clustering, Gene module discovery

## Abstract

**Background:**

Many variable selection techniques have been proposed for the clustering of gene expression data. While these methods tend to filter out irrelevant genes and identify informative genes that contribute to a clustering solution, they are based on criteria that do not consider the potential interactive influence among individual genes. Motivated by ensemble clustering, there is a strong interest in leveraging the structure of gene networks for gene selection, so that the relationship information between genes can be effectively utilized, while the selected genes are expected to preserve all the possible clustering structures in the data.

**Results:**

We present a new filter method that uses the gene connectivity in the gene co-expression network as the evaluation criteria for variable selection. The gene connectivity measures the importance of the genes in term of their expression similarity with others in the co-expression network. The hard threshold and soft threshold transformations are employed to construct the gene co-expression networks. Both simulation studies and real data analysis have shown that the network based on soft thresholding is more effective in selecting relevant variables and provides better clustering results compared to the hard thresholding transformation and two other canonical filter methods for variable selection. Furthermore, a new module analysis approach is proposed to reveal the higher order organization of the gene space, where the genes of a module share significant topological similarity and are associated with a consensus partition of the sample space. We demonstrate that the identified modules can lead to biologically meaningful sample partitions that might be missed by other methods.

**Conclusions:**

By leveraging the structure of gene co-expression network, first we propose a variable selection method that selects individual genes with top connectivity. Both simulation studies and real data application have demonstrated that our method has better performance in terms of the reliability of the selected genes and sample clustering results. In addition, we propose a module recovery method that can help discover novel sample partitions that might be hidden when performing clustering analyses using all available genes. The source code of our program is available at http://nba.uth.tmc.edu/homepage/liu/netVar/.

## Background

Variable selection in high-dimensional clustering analysis has drawn attention recently in a variety of fields, including statistics, machine learning, pattern recognition and bioinformatics. Generally, variable selection algorithms can be categorized as either wrappers or filters. In the context of clustering, the wrapper approach searches for variables best suited to a specific clustering algorithm aiming to improve the clustering performance [1,2]. The wrapper approach has been shown to be effective on low dimensional data. However, one problem for these methods, when applied to large data sets, is the increase in computational complexity as the search space exponentially increases over the number of variables. Furthermore, the wrapper method lacks robustness and is biased towards the clustering algorithm used [3]. In contrast, the filter approach is more efficient in dealing with these drawbacks. Filter-based algorithms do not involve clustering algorithms for the evaluation of variable subsets. Rather, the variables are evaluated according to certain criteria (e.g., feature variance [4], entropy-based distance [5], similarity among feature [6], Laplacian score [7]). The filter approach is considered faster and more efficient than the wrapper method in high-dimensional data analysis.

DNA microarray datasets are examples of high-dimensional data characterized by low sample sizes and high dimensionality of variables. Clustering microarray data can be very useful for biological and medical studies. For example, based on the gene expression profiles, interesting cluster distinctions can be found among groups of samples, which may correspond to particular phenotypes, such as different types of cancer [8]. In addition to the sample clustering, selecting the informative genes that best define the clusters of samples is also important. Therefore, many variable selection approaches have been proposed for the clustering analysis of microarray data, including the nonparametric density-based methods [9,10] and the parametric mixture model-based approaches [11,12]. In this context, Pan and Shen [13] employed an extra L-1 penalty term of mean vectors in the likelihood function to simultaneously perform variable selection and maximize the penalized likelihood [13]. Recently, a new sparse clustering method achieved variable selection by optimizing a weighted within cluster sum of squares (WCSS), subject to constraints on the weights, in the framework of K-Means clustering [14]. However, the results from these methods are limited and might not be able to capture the sheer complexity of gene regulation processes. While all these methods tend to identify the informative genes that contribute most to a single sample clustering solution, this clustering solution may not capture the meaningful sample partition corresponding to some phenotypes of interest. As gene expression can be influenced by many factors, such as cell type, cell differentiation, microenvironment, and external perturbation, the microarray dataset is the result of all these factors mixed together. The same set of samples can undergo different partitions according to different subsets of variables. Therefore, a good variable selection algorithm should select informative features that best preserve all the possible clustering structures in the data.

In this study, we propose a novel network-based method to achieve variable selection for microarray clustering analysis. Network analysis plays an increasingly important role in the exploration of information communication and has been used to study the information on the relationship between genes or proteins [15,16]. Here, we construct a gene co-expression network, in which nodes and edges represent genes and their expression similarity, respectively. Our proposed method is based on the assumption that each gene may induce a specific partition of the sample space in the absence of a priori information about the variable space. Therefore, given the thousands of genes in the microarray dataset, there might be thousands of distinct sample clustering solutions on the same set of samples. In this context, the imminent task is to combine these multiple partitions into a single consensus clustering, which should share as much information as possible with the given pool of sample partitions. This notion of integrating multiple clustering solutions is in line with the framework of cluster ensembles [17], which tend to reuse the existing knowledge and minimize the information loss incurred in the process of cluster assembling. Based on the premise that higher correlated gene expression profiles tend to produce more similar partition structures, we propose to assemble genes according to their expression similarity rather than their sample partitions. The objective of our work is two-fold based on the level of gene organization: first, to select a list of individual genes that shares the most amount of similarity with other genes, so that the final sample partition based on this gene list is a high-quality combination with the most consensus information among the partitions inferred by each individual gene. Intuitively, a good informative gene will have a large number of directly connected genes in the co-expression network, such that it has a strong ability of representing others. Therefore, we propose to assess genes on their connectivity in the co-expression network and to select the genes with top connectivity. The second objective of our study is to identify co-regulated subsets of genes, known as modules, which may represent different biological processes or pathways. The genes in each module are expected to be highly correlated and exhibit a coherent expression profile across samples, while others exist as background noise. Here the gene connectivity is used to further evaluate the gene topological similarity. The genes with high topological similarity with each other are identified as a gene module and should lead to a biologically meaningful sample partition. With simulation and real data analysis, we show that the gene connectivity, which measures the importance of the genes in term of their expression similarity with others in the co-expression network, is an appropriate criterion to select informative genes for sample clustering.

## Methods

### Gene co-expression network analysis

To define a measure of similarity s_ij_ between the expression profiles of genes *i* and *j*, we use the absolute value of the Pearson correlation s_ij_ = abs(cor(x_i_,x_j_)), where x_i_ and x_j_ represent the gene expression profiles for genes *i* and *j*, respectively. Therefore the similarity matrix can be denoted by S = [*s*_*ij*_], where the values of *s*_*ij*_ lie between 0 and 1. However, since microarray gene expression data are typically quite noisy, directly employing the similarity matrix for gene co-expression network analysis may not be appropriate. We find it useful to employ the following transformations that map a similarity matrix into an adjacency matrix. The first transformation is the signum function which implements hard thresholding. Specifically,

(1) $$a_{\mathrm{ij}}=\mathrm{signum}\left(s_{\mathrm{ij},}\tau \right)=\left\{\begin{array}{c} 1\;\mathrm{if}\;s_{\mathrm{ij}}\geq \tau \\ 0\;\mathrm{if}\;s_{\mathrm{ij}}<\tau \end{array}\right)$$

Here a_ij_ is an element of the adjacency matrix and s_ij_ is an element of the similarity matrix. In hard thresholding, the value of the parameter τ determines the number of genes and edges included in the resulting unweighted network. Typically, an arbitrary value of τ is chosen to exclude spurious edges, but this may lead to a loss of information. To address this issue in hard thresholding, a “soft” power transformation function has been proposed [18]:

(2) $$a_{\mathrm{ij}}\;=\;\left|\;s_{\mathrm{ij}}\;\left|{}^{\beta }\right)\right)\;$$

with a single parameter *β*, where *β* > =1. Soft thresholding results in a completely connected network with each edge being assigned a weight.

### Gene connectivity and variable selection

With the n × n symmetric adjacency matrix, the connectivity (node degree) *k*_*i*_ of gene *i* is define by

(3) $$k_{i}\;=\;\sum_{j\neq i}a_{\mathrm{ij}}$$

For the hard thresholding transformation, the connectivity of gene *i* simply equals to the number of genes that it is directly connected to in the unweighted network. For the soft thresholding transformation, the connectivity of gene *i* equals the sum of weights between gene *i* and all other genes in the weighted network. To select the relevant genes for microarray clustering analysis, we first rank the genes according to their connectivity. For the hard thresholding transformation, the genes with connectivity of 0 are removed from the original network. Therefore, the gene connectivity ranking is only applicable on the set of genes included in the resulting unweighted network, which is of a reduced size depending on the value of the threshold τ. In the weighted network, the ranking can be obtained for all the genes. Finally, the genes with low ranks are filtered out, while the genes with top ranks are considered to have high degree of connectivity and are selected for clustering analysis.

### Module identification

Our module identification method is based on the node similarity measure of their relative interconnectedness coupled with the hierarchical clustering method. Instead of using the gene correlation coefficients directly as the similarity measure, we calculate the Jaccard similarity coefficient *J*_*ij*_ based on the gene connectivity in the transformed network.

(4) $$J_{\mathrm{ij}}\;=\;\frac{h_{\mathrm{ij}}}{k_{i}+k_{j}-h_{\mathrm{ij}}}$$

Where *h*_*ij*_  =   ∑ _*u*_*a*_*iu*_*a*_*uj*_, which equals the number of genes to which both *i* and *j* are connected in the case of hard thresholding, and the total interconnectedness of genes *i* and *j* in the soft thresholding transformation. And *k*_*i*_  =   ∑ _*u*_*a*_*iu*_ is the node connectivity as defined in equation (3). Therefore, the similarity measure will be affected by the selection of the transformation parameters. In our implementation, we adjust the hard thresholding parameter τ or the power function parameter *β* to explore their effects on the results of module identification. Once the topological similarity measure matrix is obtained, we re-order it by hierarchical clustering of each row and column to put similar genes in an adjacency zone [19]. Since the similarity measure matrix is symmetric, these highly similar genes would form “hot” blocks along the diagonal and can be identified as a module by visual inspection. The genes in the resulting modules are expected to be highly co-expressed.

### Evaluate the performance of variable selection

The performance of our method for variable selection is evaluated by the F-score, where F = 2*Precision*Recall/(Precision + Recall). The precision is the proportion of selected variables that are truly relevant, and the recall is the proportion of truly relevant variables that are selected by our method, also known as the true positive rate. The F-score ranges between 0 and 1, and can be interpreted as a weighted average of the precision and recall.

Based on the selected genes, we cluster samples using the K-means algorithm with 50 iterations. The sample clustering performance is evaluated by the classification error rate (CER). The derived sample clustering (p_1_) is compared to the true clustering (p_2_) to assess the performance. The CER is defined as

(5) $$\mathrm{CER}\left(p_{1},p_{2}\right)\;=\frac{\sum_{i>i'}\left|1_{p_{1}\left(i\right)==p_{1}\left(i'\right)}-1_{p_{2}\left(i\right)==p_{2}\left(i'\right)}\right|}{\left(\begin{array}{l} n\\ 2 \end{array}\right)}$$

where n is the sample size. Note that smaller CER values reflect more accurate clustering results. A CER of zero indicates that the clustering results p_1_ and p_2_ agree perfectly.

### Simulation data setting

We used a simulation setting similar to that in Witten and Tibshirani [14]. A simulated dataset contains 60 samples from three classes C_1_, C_2_ and C_3_ (20 samples from each) and each sample *X*_*i*_ is a d - dimensional vector that follows *N*(μ_*i*_, ∑ _*d*_) and is independent of other samples. Thus, the clustering structure is determined by the specification of μ_i_’s that are defined as

(6) $$\mu_{\mathrm{ij}}=\left\{\begin{array}{rr} \mu \left(1_{i\in C1}‒1_{i\in C\backslash 1}\right) & \mathrm{if}\;j\leq 10\\ \mu \left(1_{i\in C\backslash 1}‒1_{i\in C1}\right) & \mathrm{if}\;10<j\leq 20\\ \mu \left(1_{i\in C2}‒1_{i\in C\backslash 2}\right) & \mathrm{if}\;20<j\leq 30\\ \begin{array}{l} \mu \left(1_{i\in C\backslash 2}‒1_{i\in C2}\right)\\ 0 \end{array} & \begin{array}{l} \mathrm{if}\;30<j\leq 40\\ \;\mathrm{otherwise} \end{array} \end{array}\right)$$

Where μ is a positive constant and set to 1 in the experiment. This configuration of μ sets the first 40 genes as informative genes and the other genes as noise. We take ∑ _*d*_  =  *diag* (*σ*_1_, …, *σ*_*d*_) where fc *σ*_1_, …, *σ*_*d*_ are set such that the population variance of each variable is one. In the simulation, the first 20 genes together can be considered as a module since their expression profiles are highly correlated and this module differentiates samples in class C_1_ from the others. Whereas the next 20 genes form another module that differentiates samples in class C_2_ from others. Therefore, these two sets of genes exhibit different sample partitions.

## Results and discussion

### Variable selection performance with simulation dataset

**Figure 1:**
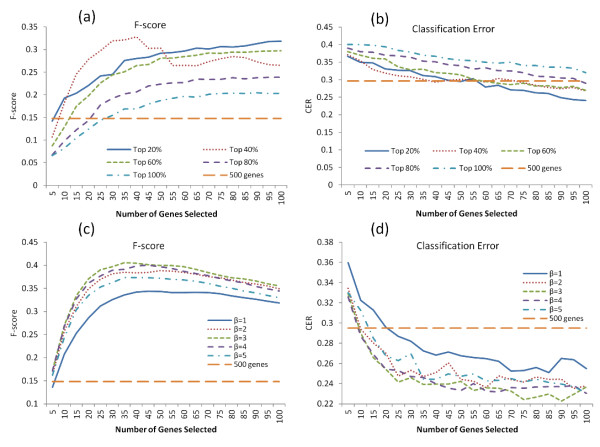
**Performance of variable selection.** The averaged F-scores **(a, c)** and the CER curves **(b, d)** in hard thresholding and soft thresholding transformation, respectively. The horizontal line in each plot represents the performance based on all genes (500 totally).

We describe herein the performance of our method with two network inference methods on a simulated dataset. In the hard thresholding transformation, we considered the effects of two parameters on the performance of variable selection: the hard threshold τ that determines the number of genes and edges included in the unweighted co-expression network, and the percentage of genes to be selected based on their connectivity in the resulted network, determined by equation (3). With a greater value of τ, the resulting network will have a smaller number of genes and edges, but the connection strength (correlation coefficient) between paired genes will be higher. We reported the average F-scores and CER values based on 100 simulated datasets for two different dimensionalities (d=500, Figure 1a and b, and d=1000, Additional file 1: Figure S1a and b).

It is not surprising to observe that selecting all of the 500 genes in the dataset can only lead to a low F-score (0.15) and a high CER (0.29), as shown in Figure 1a and b, simply because too many noninformative genes were included without the variable selection step. When we varied the threshold τ to generate a network with reduced size but kept all the genes in the resulting network regardless of their connectivity (in the case of genes with top 100% connectivity being selected), the performance evaluated by the F-score was improved but still poor, regardless of how many genes were in the resulting network. However, both the F-scores and CER were shown to improve further with an additional step of gene filtering by the gene connectivity rank. Generally, the more stringent the gene connectivity rank filtering, the lower the number of genes selected. To compare the performance of different gene connectivity ranks with the same number of genes selected, we had to decrease the threshold τ to achieve a large network size when the gene connectivity rank was more stringent. As shown in the Figure 1a and b, the gene filtering with the top 20 percentile connectivity resulted in the highest F value and the lowest CER when 100 genes were selected. However, this was not always the case. When 40 genes were included, the top 40% percentile rank achieved best performance among all these filtering scales. This indicates that the performance of variable selection is affected by both the connection strength and the connectivity of the selected genes. We observed similar results in both simulated datasets with different dimensionalities (d= 1000, Additional file 1: Figure S1a and b).

As shown in above analysis, the network variable selection based on hard-thresholding transformation was influenced by both the network size and the gene connectivity filtering. It may be challenging to optimize both of these two factors in real data analysis. To resolve this problem, we used a soft thresholding transformation for gene selection that is only dependent on the power function parameter *β*. It not only takes into account the information of all the genes, but also reduces the effect of noise induced correlation by the power function, assuming that the noise correlation occurs more likely at smaller values than the correlation associated with true gene relationships.

In the soft thresholding transformation, we varied the value of *β* to construct a series of gene co-expression networks. Results in Figure 1c and d demonstrated that the power transformation significantly improved the performance of variable selection and led to a higher F-score peak and lower CER than the original non-transformed one (*β*=1). We further found the performance was not a monotonic function of *β*. Among the four power functions with different parameters *β*, the optimal value of F-score and CER were achieved when *β* was set to 3, which may result in the optimized state for emphasizing the correlation associated with true gene relationships by diminishing the noisy effects in this simulation setting. We observed similar results when d=1000 (Additional file 1: Figure S1c and d).

### In comparison with other feature selection methods

**Figure 2:**
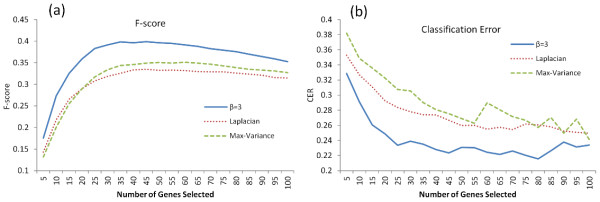
**Comparison with other variable selection methods. (a)** The averaged F scores and **(b)** the CER curves. Our method is based on soft thresholding transformation with the power function parameter β=3.

To further demonstrate the effectiveness of our proposed network based analysis for variable selection, we compared it with two other classic filter algorithms, the Laplacian Score [7] and the Max-Variance. As a special case of a spectral feature selection algorithm, Laplacian score selects those features that can best preserve the local manifold structure (He et al. [7]). In the comparison, we set up the parameter *β* with the optimized value obtained in the aforementioned simulation result (*β* =3). The genes selected from each method were used for clustering samples with the K-means algorithm. Our proposed network algorithm consistently outperformed the other two methods, particularly at a low dimensionality (20–100), where all of these methods reached their best performance (Figure 2b). The Laplacian score method achieved an optimal CER value of 0.25, close to that of the network without a power transformation. Similar results were observed in the comparison of the F-scores (Figure 2a). This indicates the effectiveness of our variable selection method in clustering analysis.

### Selecting genes that support a common clustering structure by module identification

The gene co-expression network analysis captures the relationships among the genes so that it can help identify a small number of sets of highly correlated genes, each of which tends to assemble into a functional module that can be involved in biological pathways or molecular complexes. Also, these genes together assure a specific clustering of samples, which might be different from other sets of correlated genes. The genes selected by a module usually have greater intramodular connectivity than those that do not belong to the module. Therefore, module analysis not only captures the connectivity information of individual nodes as what we did in the variable selection, but also reveals the higher order organization of gene topological similarity in the entire gene space.

**Figure 3:**
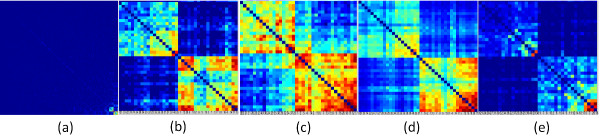
**Module structure in the gene co-expression network from the simulated dataset of d = 500. (a)**. Two modules were identified in the co-expression network. The rest on the right are zoomed-in view of the modules highlighting the genes included in two modules respectively. **(b)** and **(c)**, hard thresholding transformation, with top 1% and 5% correlations included in the network, respectively. **(d)** and **(e)**, soft thresholding transformation, with the power functions parameter β=3 and β=7, respectively.

We applied both the hard-thresholding and soft-thresholding transformation on the gene co-expression network for module identification. Note that the sensitivity of this method varies depending on the co-expression network size and the composition of variable space. In the analysis, we assigned the value of μ to 1.5 in the simulation setting to demonstrate a clear module structure. For hard thresholding transformation of network (d=500), we varied the threshold τ to retain the top 1% of correlation coefficients for calculating the Jaccard similarity coefficients between genes. This cutoff is roughly consistent with our simulation setting where there are 40 informative genes and 40*40 informative gene pairs among 500*500 total number of gene pairs (0.64%). We also tested the performance on the top 5% of correlations. Figure 3b and c showed the discovered modules in the network when the top 1% and top 5% of correlations were kept in the transformed network, respectively. Two ‘hot blocks’ can be clearly identified along the diagonal, each of which corresponding to the original defined module in the simulation setting with only a few missing genes. Due to varying numbers of edges included, the boundaries between blocks exhibited distinctive sharpness in Figure 3b and c, but the module structure and the genes included in each module were the same. Therefore, this simulation result can serve as a guideline for determining cutoffs. For a hard threshold of 1%, we are assuming that roughly 10% of the genes are informative and 90% are not. This assumption is of course not optimal for every dataset. Fortunately, this simulated example suggests that module identification is not very sensitive to this parameter. Therefore, in a real dataset, if we use a hard threshold, we will first set the threshold to select the top 1% of edges, and also vary the threshold slightly, while checking whether the hot block appears to be consistent with respect to small changes of the cutoff.

We also performed soft thresholding transformation and obtained similar results (Figure 3d and e), indicating the relative robustness of our method in module identification. Furthermore, each of the blocks induced a unique bipartitioning of the sample space that is equivalent to the sample partition inferred by the corresponding modules in our simulation setting (C1 vs. others and C2 vs. others). We observed similar results when d=1000 (Additional file 1: Figure S2).

### Application in real datasets

Along with simulations, we applied our method to two real experimental datasets: Leukemia [8] and Colon cancer data [20].

The leukemia dataset consists of 72 patients with two subtypes of acute leukemia: acute myeloid leukemia (AML) and acute lymphoblastic leukemia (ALL). The latter is composed of two subclasses, B-cell and T-cell types. Therefore there could be two possible biologically meaningful clustering solutions including one with two-clusters of samples (AML and ALL) and the other with three clusters (T cell ALL, B cell ALL and AML).

**Figure 4:**
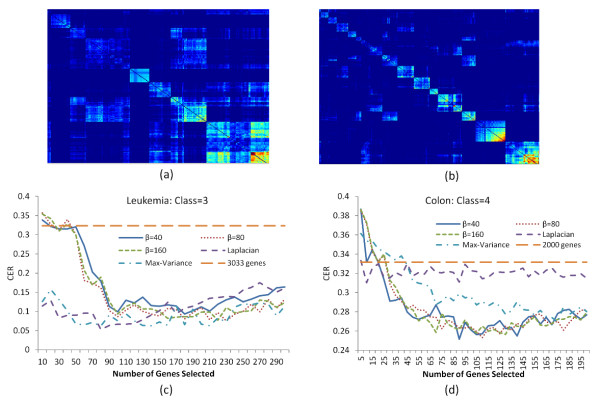
**Module analysis and clustering results for the leukemia and colon datasets.** Top panel: Zoomed-in view of the module composition in the gene co-expression matrices of the Leukemia **(a)** and Colon dataset **(b)**. Bottom panel: The CER curves based on soft thresholding transformation with various power functions for three clusters of Leukemia dataset **(c)** and four clusters of Colon cancer dataset **(d)**.

**Figure 5:**
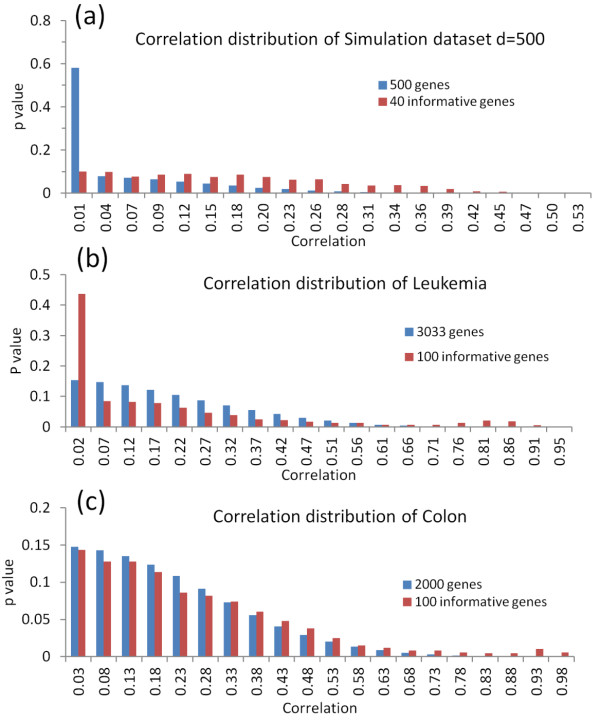
**The correlation distribution of full variable space and informative gene set. (a)** The simulated dataset with d=500, **(b)** the Leukemia and **(c)** the colon cancer dataset.

Following Dudoit and Fridlyand [21], three pre-processing steps were applied to the original data matrix and a final 72 X 3571 data matrix was obtained. Because the pre-processing steps included thresholding the gene expression values with a floor and a ceiling boundaries, many artificially high correlations were introduced. We filtered out these genes whose medians equal the boundary values and obtained 3033 genes totally. We first studied the module organization of the gene space and the associated sample partition in the leukaemia dataset. In the implementation, the value of τ was chosen to include only the edges corresponding to the top 1% of paired correlation coefficients in the network. As shown in Figure 4a, the topological similarity matrix exhibited a sharp separation of modules from its neighboring genes. We evaluated the sample clustering performance of modules by using the gene set included in each module for sample partitioning. We found that most of them induced a meaningful partition of the sample space. Specifically, the first module at the bottom right corner rendered a dichotomy of the samples according to the known classification, ALL/AML, with the CER value equalling 0.155, whereas the second module tends to distinguish B cell ALL patients from the rest with a CER value of 0.2, indicating the unrecognized similarity between T cell ALL samples and AML samples in the dataset. The other modules also imposed a potential novel partition of samples. These results confirmed multiple possible clustering solutions in the leukemia dataset. We also performed variable selection to select individual genes based on their connectivity in the transformed network using soft thresholding transformation. For the three-cluster solution, the 100 genes selected from the network-based analysis yielded the sample partition coinciding almost precisely with the known classification (T cell ALL, B cell ALL and AML) with CER equalling to 0.09, when the parameter β reached 40 or above (Figure 4c). In Figure 4c, we also compared the performance of our network-based method with other filter methods. Results showed that our approach achieved a comparable optimal CER value with the Laplacian and the Max-Variance methods, when more than 100 genes were selected. Out of the top 100 informative genes selected from our method, 28 and 35 genes were found by the Laplacian and the Max-Variance method, respectively (Additional file 1: Figure S4a). The genes selected from our method may also represent new sample partitions. This is supported by the observation that our method achieved better separation of B cell AML samples from the rest compared to other methods (Additional file 1: Figure S5a). We further examined the gene lists selected from soft thresholding transformation with varied β, and found that the overlap among the gene lists increased as β became larger. After β reached 80, the selected gene lists were almost unchanged. This is consistent with their similar clustering performance as shown in Figure 4c. However, recall that in our simulation studies the optimal CER was achieved when β was small (β **=**3), reflecting possibly different variable compositions between the simulated dataset and the leukemia data. As shown in Figure 5a and b, these implications became clear. In Figure 5a, the correlation of 40 informative genes in the simulated dataset followed a uniform distribution. Unlike the 40 informative genes in simulation, the correlation of the 100 selected informative genes from the leukemia data followed a mixture distribution with two components, one at the high end of the distribution and another close to zero (Figure 5b). This is reasonable for real datasets given the assumption that gene expression data are influenced by many active biological processes, where genes within each of the processes tend to be highly correlated with one another, but may not be well correlated with those participating in other biological processes. Therefore, the correlation values between genes corresponding to different biological processes will be small, located in the low end of the correlation distribution. Since the component in the high end was well separable from the other for the full gene space, the corresponding highly connected genes tend to be always selected as informative genes disregarding the value of β. Therefore, the selected genes should not be sensitive to β when β is above a certain threshold that aims to remove the noisy correlations. However, this was not the case for the simulated dataset, where the correlation of the informative genes followed a uniform distribution while that of the full set of genes was close to zero, so a small value of β should be able to remove the noise induced correlations.

We also analyzed the colon cancer data by Alon et al. [20], which contains two classes of samples based on disease status: 40 tumor samples and 22 normal samples. In addition, an independent study reported that there was a difference in the experimental protocols used to process the samples [22]. There are 22 samples processed by protocol 1, and the other 40 samples were processed by protocol 2. Taking the different protocols into consideration, the study has at least three different possible sample partition structures based on disease status, sample protocols, and their combination. In the analysis, we first employed the module analysis on the dataset, followed by variable selection for the clustering analysis. As we did with the leukemia dataset, the edges corresponding to the top 1% correlation were kept for module analysis. As shown in Figure 4b, dozens of modules were identifiable along the diagonal of the similarity matrix. Each module exhibited a distinctive partition of the sample space. Among the first three modules at the bottom right, module 1 had strong tendency to partition the samples according to the normal versus tumor classification with a CER value (0.35), whereas module 3 was informative for the partition based on different protocols (CER=0.27). Also the number of genes included in these two modules differed. Module 1 was the largest in terms of the number of genes included. These results indicate that the classification of tumor versus normal samples is a more dominant factor in the sample clustering compared to the different sample protocols. It was interesting to observe that the number of genes in module 2 was similar compared to module 1. However their clustering behaviours differed, suggesting that module 2 may inform a novel sample partition of the colon cancer dataset. The aforementioned module analysis reinforced the idea that the colon cancer dataset has at least three clustering solutions. Here, a soft thresholding transformation was implemented to select the feature genes. As shown in Figure 4d, in a four-cluster solution that combines the disease status and the sample protocol changes, the clustering performance of our method based on different values of β is similar given a β value of 40 or above. Here, the CER value was higher than that obtained from the leukemia dataset, possibly due to mislabeled samples [23]. Nevertheless, our approach achieved a better performance than the other methods. In Figure 4d, when β was 40 or above, our method had a CER value equal to 0.25 with 100 genes selected, whereas Laplacian and Max-Variance obtained the CER values of 0.32 and 0.29, respectively. Only 18 and 8 genes selected from our approach were also obtained from the Laplacian and the Max-Variance method, respectively (Additional file 1: Figure S4b). For the other clustering solution based on different experimental protocols, our method outperformed others as well (Additional file 1: Figure S5b). We also plotted the distribution of correlations for full gene space and the 100 selected informative genes respectively (Figure 5c). Similar to the leukemia dataset, the gene set has two well-separated components of correlations at two ends, which may explain their saturating behaviour of clustering performance when β reaches a certain value.

## Conclusions

Variable selection for clustering is never a trivial problem. This is particularly true in high dimensional data analysis, where few dozens of informative variables are often dispersed over a noisy background with thousands of noninformative variables. Traditional approaches to filtering out the irrelevant features are based on certain criteria that do not account for potential interactive influence from other individual variables. Motivated by ensemble clustering, we propose a new filter score, the gene connectivity in the co-expression network, which takes into account all of the information gained from other nodes in the network in terms of expression similarity; therefore, the selected genes are expected sustain a consensus sample partition that populates through the partition pool induced by individual genes.

To obtain the connectivity of each gene, we have applied two network inference methods based on a hard thresholding and a soft thresholding adjacency function. In the first method, we use a hard threshold parameter τ to infer the gene network, followed by filtering the nodes based on their connectivity rank. Therefore, the resulted feature gene set is affected by the resulting network size and the stringency of the connectivity rank. The marginal gene connectivity obtained from hard thresholding transformation is estimated solely based on the given gene and its neighbourhood in the network with reduced size. Therefore, to retain more information of the entire network, we employ the soft thresholding transformation to build a complete network including all genes, where each gene is connected to all the other genes in the network with weighted connection strength. Our simulation results showed that soft thresholding is more effective and provides better clustering results compared to the hard thresholding method in terms of clustering error rate and variable selection. We realize although the connectivity obtained from a soft thresholding network preserves more information of the entire network compared to the hard thresholding transformation, the gene connectivity calculation is still based on the gene and its edges to all the genes in the network, and it does not consider the edges among its neighbors. There are other network related metrics, such as the betweenness centrality of a node that requires knowledge of the entire network topology and indicates how important the node is within the context of the entire network. These network metrics will be evaluated in our future studies. In this work, considering the fact that gene connectivity is easy to calculate and the derived gene hubs represent a large number of other genes in the network well, we use gene connectivity as our evaluation criteria for variable selection.

The parameter setting in variable selection methods renders a crucial influence on the performance of the feature genes selected. However, tuning parameter selection in the unsupervised setting is always a challenging problem [24]. In real data analyses, we showed the relative robustness of our method when the parameter β was above a threshold value in the soft thresholding transformation. Further analysis of these informative genes demonstrates a mixture correlation distribution with two components in the profile. The component in the high end comes from paired gene correlations within the same biological processes, so they will be preferentially selected by the high power transformation parameter β compared to other genes. Therefore, we propose to pick the value of β for a given dataset so that the selected genes are not sensitive to β if it is above this threshold value. Alternatively, we can evaluate each set of feature genes with other criteria, such as the purity and efficiency of clustering results, or within/between class distance of induced sample partitions.

Furthermore, we developed a module identification method by measuring the node interconnectedness in the co-expression network. Our module identification is based on using a node similarity measure in conjunction with a clustering method. In this study, we chose to use the Jaccard similarity coefficient based on the gene connectivity instead of using the gene correlation coefficients directly. Jaccard similarity takes advantage of the co-expression network information assuming that two nodes having a higher degree of overlapping neighbors are more likely to be in the same functional class than nodes having a lower topological overlap. This is particularly useful when high background noise divert the real network information. We performed a comparison of module structures based on two similarity measures: the Pearson correlation coefficient and the Jaccard similarity coefficient. As shown in Additional file 1: Figure S3, there is a clear difference before and after the Jaccard similarity was computed on the gene correlation coefficients. The Jaccard similarity coefficient led to more distinct gene modules than the correlation coefficient which resulted in a highly noisy background on its module structure. Once the node similarity measure is obtained, we applied hierarchical clustering to resort the rows and columns of genes. There are alternative clustering procedures such as K-means clustering, but hierarchical clustering is more straightforward, and does not require specification of the number of modules. The genes corresponding to each module clearly form squares along the diagonal so that the modules can be easily identified by visual inspection.

The focus in our module analysis is on the high order organization of gene space, rather than their specific corresponding sample partitions. It is particularly useful for discovering unanticipated sample partition structures in data. Unlike the previous methods [22], our method does not need the partitioning and merging steps of the gene space; alternatively, we use the co-expression network, which is capable of recovering biologically meaningful modules amongst a noisy background, that putatively represent pathways or cellular processes. Such information can be used to establish causal models connecting the informative feature sets with known phenotypes such as disease symptoms, which will facilitate discovery of new and hidden patterns in datasets.

## Competing interests

The authors declare that they have no competing interest.

## Authors’ contributions

ZW carried out the simulation studies and real data application and drafted the manuscript. FAS participated in the data analysis and results discussion. QP participated in the design of the study and helped to draft the manuscript. YL conceived and coordinated the study and helped to draft the manuscript. All authors read and approved the final manuscript.

## Supplementary Material

Additional file 1: Figure S1 Variable selection performance in the simulated dataset (d= 1000). The averaged F-scores (a, c) and the CER curves (b, d) in hard thresholding and soft thresholding transformation, respectively. The horizontal line in each plot represents the performance based on all genes (1000 totally). **Figure S2.** Module structure in the gene co-expression network from the simulated dataset of d=1000. (a) and (b): in hard threshold transformation, with top 1% and 5% correlations were included in the network, respectively. (c) and (d): in soft transformation, power function with β=3 and β=7. **Figure S3.** Comparison of module structure recovery using different similarity measures. The rows and columns of genes have been reordered according to the hierarchical clustering of similarity matrix. (a). Pearson correlation coefficients of 500 genes, no transformation. (b) Jaccard similarity coefficients of 500 genes, no transformation. (c) Pearson correlation coefficients with power transformation with β=3. (d). Jaccard similarity coefficients derived from power transformation with β=3. **Figure S4.** Comparison of lists of genes selected from different methods. Network refers to our network-based variable selection method. (a) Leukemia dataset and (b) Colon cancer dataset. **Figure S5.** Clustering performance based on new partition structures identified by our module analysis. The CER curve with various power functions in co-expression network transformation for new partition structures of the Leukemia dataset (a) and Colon dataset (b). Click here for file
