# Supplementary material for: Improving the sensitivity of sample clustering by leveraging gene co-expression networks in variable selection
Source: BMC Bioinformatics. 2014 May 20;15:153. doi: 10.1186/1471-2105-15-153 (PMC4035826; doi:10.1186/1471-2105-15-153)
Supplement: Additional file 1: Figure S1 — Variable selection performance in the simulated dataset (d= 1000). The averaged F-scores (a, c) and the CER curves (b, d) in hard thresholding and soft thresholding transformation, respectively. The horizontal line in each plot represents the performance based on all genes (1000 totally). Figure S2. Module structure in the gene co-expression network from the simulated dataset of d=1000. (a) and (b): in hard threshold transformation, with top 1% and 5% correlations were included in the network, respectively. (c) and (d): in soft transformation, power function with β=3 and β=7. Figure S3. Comparison of module structure recovery using different similarity measures. The rows and columns of genes have been reordered according to the hierarchical clustering of similarity matrix. (a). Pearson correlation coefficients of 500 genes, no transformation. (b) Jaccard similarity coefficients of 500 genes, no transformation. (c) Pearson correlation coefficients with power transformation with β=3. (d). Jaccard similarity coefficients derived from power transformation with β=3. Figure S4. Comparison of lists of genes selected from different methods. Network refers to our network-based variable selection method. (a) Leukemia dataset and (b) Colon cancer dataset. Figure S5. Clustering performance based on new partition structures identified by our module analysis. The CER curve with various power functions in co-expression network transformation for new partition structures of the Leukemia dataset (a) and Colon dataset (b). [file 1471-2105-15-153-S1.docx]

**Improving the Sensitivity of Sample Clustering by Leveraging Gene Co-Expression Networks in Variable Selection**

### Zixing Wang^1^, F. Anthony San Lucas^2,4^, Peng Qiu^3^ and Yin Liu^1,4§^

^1^Department of Neurobiology and Anatomy, University of Texas Health Science Center at Houston, Houston, Texas, United States of America

^2^Department of Epidemiology, University of Texas MD Anderson Cancer Center, Houston, Texas, United States of America

^3^Department of Biomedical Engineering, Georgia Institute of Technology, Atlanta, Georgia, United States of America

^4^University of Texas Graduate School of Biomedical Sciences, Houston, Texas, United States of America

^§^Corresponding author

Email addresses:

ZW: [zixing.wang@uth.tmc.edu](mailto:zixing.wang@uth.tmc.edu)

FAS: sanlucas@gmail.com

PQ: pqiu7@mail.gatech.edu

YL: yin.liu@uth.tmc.edu


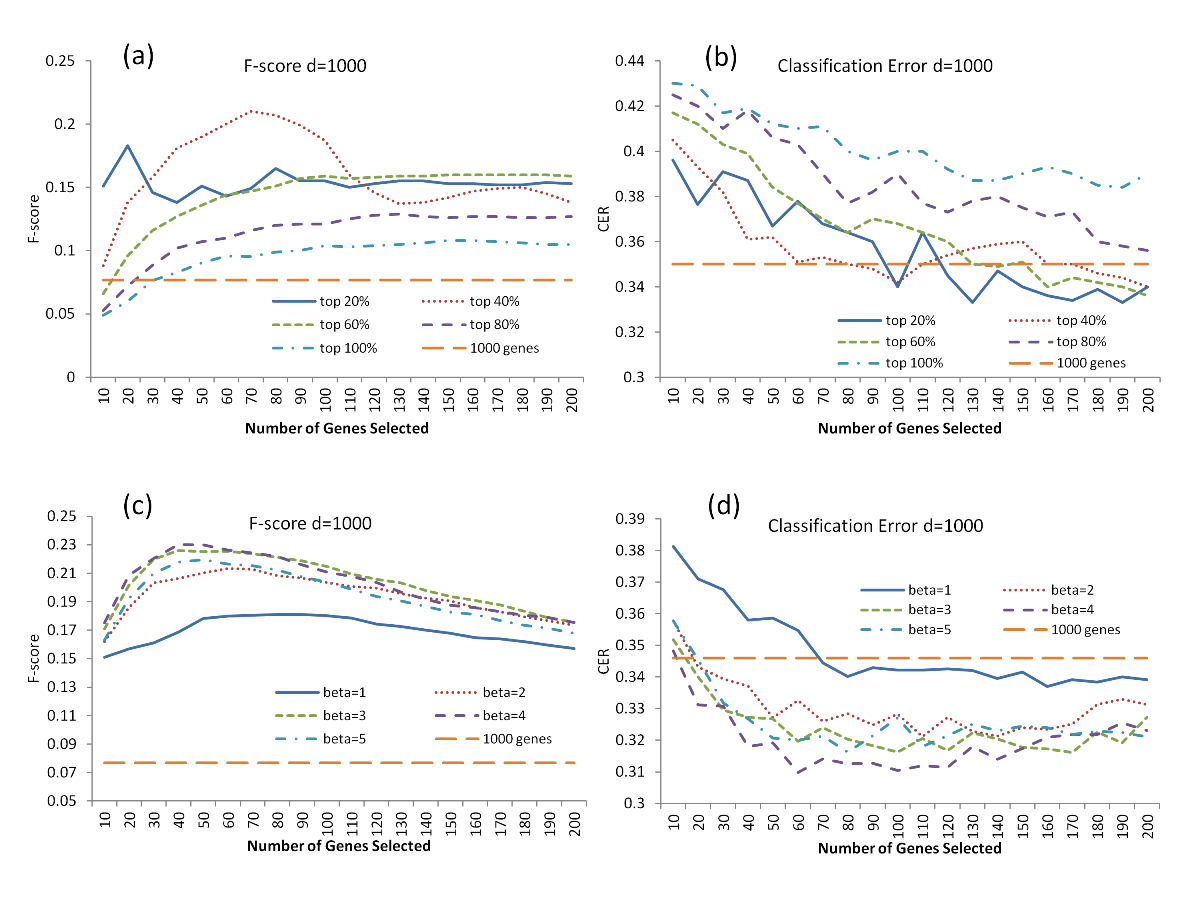


## Supplementary Figure 1. Variable selection performance in the simulated dataset (d= 1000). The averaged F-scores (a,c) and the CER curves (b,d) in hard thresholding and soft thresholding transformation, respectively. The horizontal line in each plot represents the performance based on all genes (1000 totally).

###
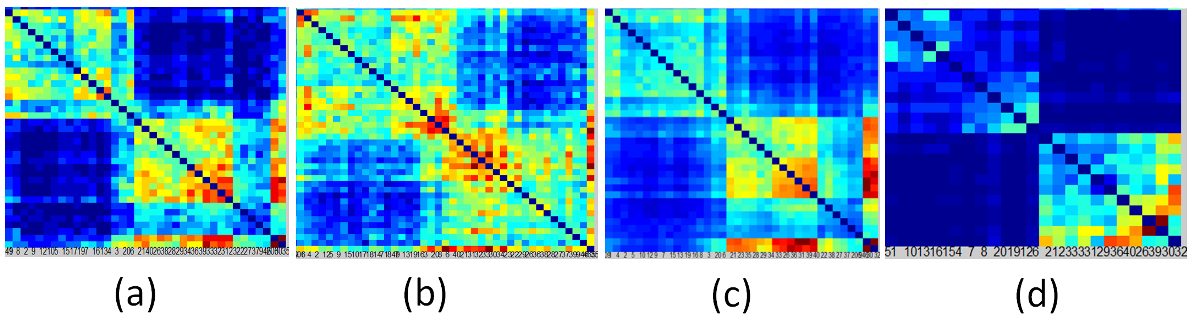


## Supplementary Figure 2. Module structure in the gene co-expression network from the simulated dataset of d=1000. (a) and (b): in hard threshold transformation, with top 1% and 5% correlations were included in the network, respectively. (c) and (d): in soft transformation, power function with β=3 and β=7.

**
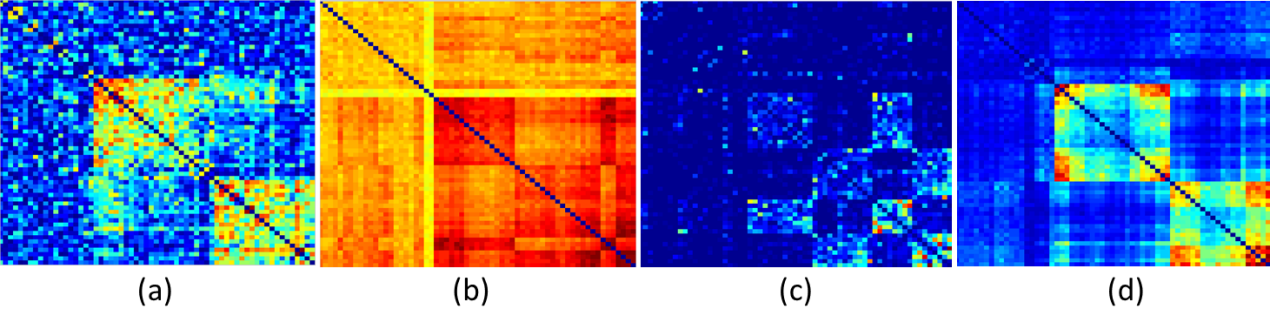
**

**Supplementary Figure 3.** **Comparison of module structure recovery using different similarity measures.** The rows and columns of genes have been reordered according to the hierarchical clustering of similarity matrix. (a). Pearson correlation coefficients of 500 genes, no transformation. (b) Jaccard similarity coefficients of 500 genes, no transformation. (c) Pearson correlation coefficients with power transformation with β=3. (d). Jaccard similarity coefficients derived from power transformation with β=3.


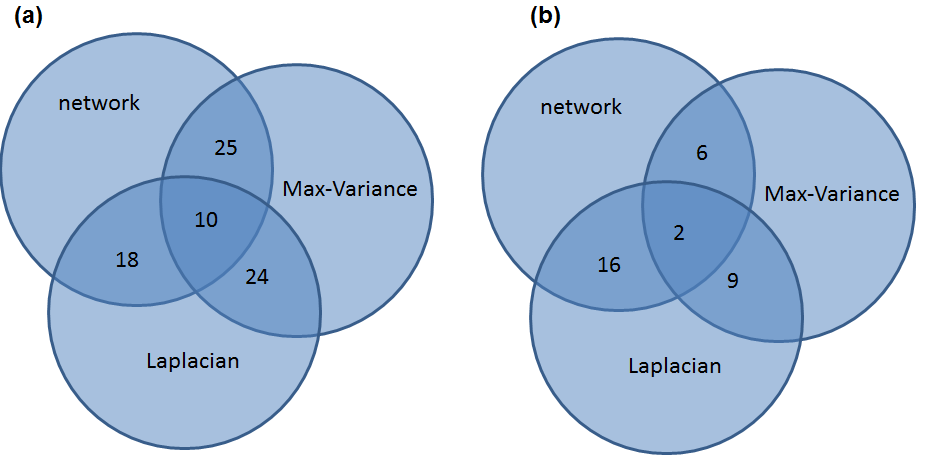


**Supplementary Figure 4. Comparison of lists of genes selected from different methods.** Network refers to our network-based variable selection method. (a) Leukemia dataset and (b) Colon cancer dataset.

###
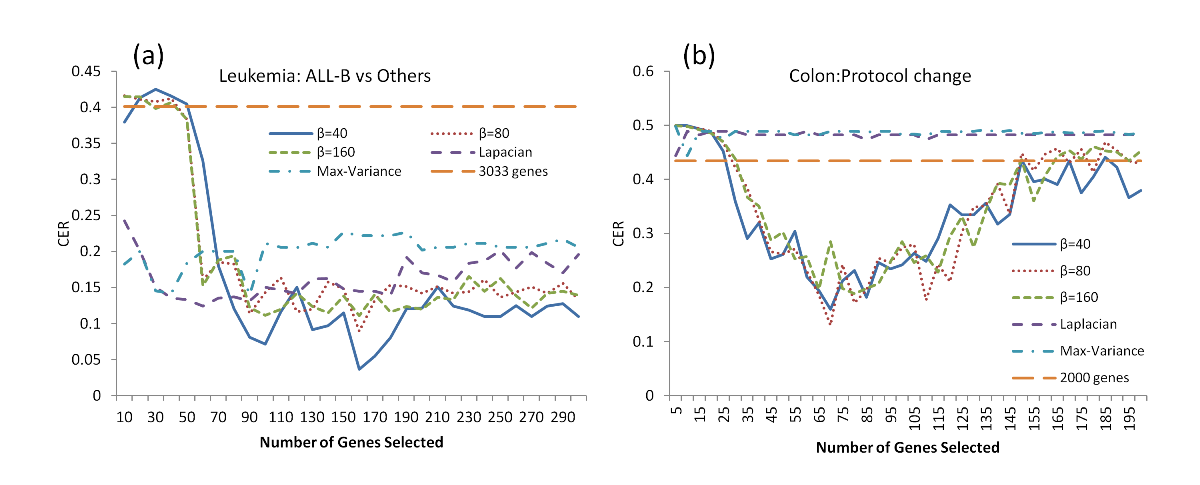


**Supplementary Figure 5. Clustering performance based on new partition structures identified by our module analysis.** The CER curve with various power functions in co-expression network transformation for new partition structures of the Leukemia dataset (a) and Colon dataset (b).
